# Volvulus on a Giant Cystic Lymphangioma: Two Cases of the Rare Cause of Intestinal Obstruction in Children

**DOI:** 10.7759/cureus.85578

**Published:** 2025-06-08

**Authors:** Amara Ayoub, El Atrache Hanae, Zaari Najlae, Abdelouhab Ammor, Houssain Benhaddou

**Affiliations:** 1 Faculty of Medicine and Pharmacy, Mohamed First University, Oujda, MAR; 2 Department of Pediatric Surgery, Mohammed VI University International Hospital, Oujda, MAR; 3 Department of Pediatric Surgery, Centre Hospitalier Universitaire Mohammed VI, Oujda, MAR

**Keywords:** cystic lymphangioma, jejunal volvulus, paediatric intestinal obstruction, primary mesenteric cyst, rare volvulus

## Abstract

Cystic lymphangiomas (CLs) are rare benign vascular malformations, primarily occurring in children, often in the cervicofacial region. Intra-abdominal lymphangiomas, though less common, can lead to serious complications such as volvulus and intestinal obstruction. We report two cases of volvulus resulting from mesenteric CLs (MCLs) in pediatric patients. Both patients presented with acute intestinal obstruction and underwent emergency laparotomy, which included complete resection of the cysts, resulting in favorable postoperative outcomes. These cases underscore the necessity of considering CLs in the differential diagnosis of pediatric abdominal masses and the importance of prompt surgical intervention to prevent severe complications. Additionally, we discuss the pathophysiology, imaging techniques, surgical management, and histopathological findings associated with this rare condition.

## Introduction

Mesenteric cystic lymphangiomas (MCLs) are rare congenital malformations of the lymphatic system, with an estimated incidence of one in 250,000 pediatric hospital admissions. These cystic structures arise due to abnormal lymphatic vessel development and are often diagnosed in early childhood. While most lymphangiomas occur in the cervicofacial region, intra-abdominal involvement represents only 5-10% of cases. Complications such as volvulus, hemorrhage, and infection necessitate prompt diagnosis and intervention [1-3].

We report two pediatric cases of volvulus caused by mesenteric cystic lymphangioma, emphasizing the diagnostic challenges, imaging findings, surgical approach, and postoperative outcomes.

## Case presentation

Case 1

A two-year and six-month-old male child was admitted to the emergency department with a four-day history of abdominal distension, bilious vomiting, and absence of stool and gas passage. The child was hemodynamically stable but presented with signs of dehydration. Clinical examination revealed a distended, tender abdomen without palpable masses.

Imaging Findings

**Figure 1:**
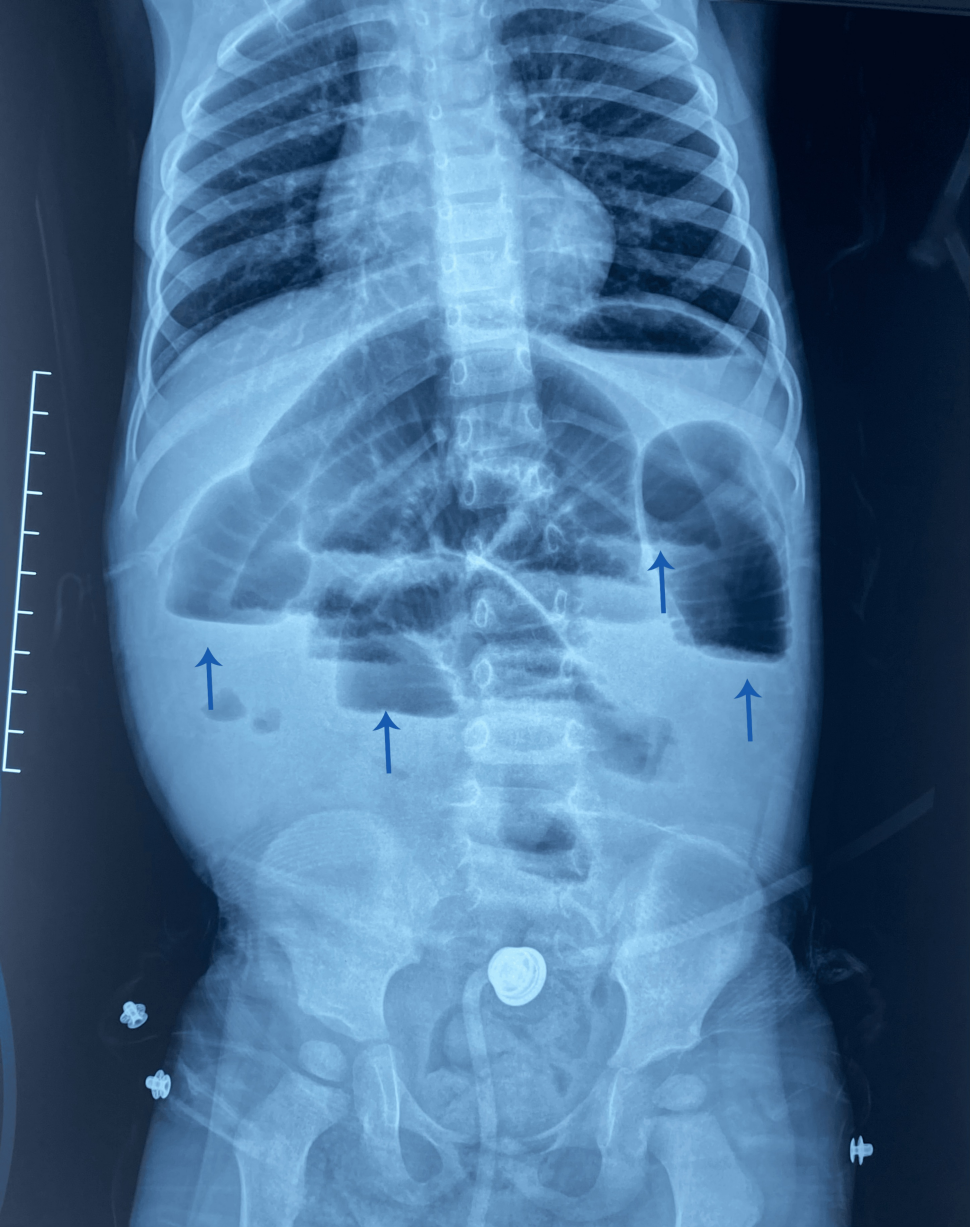
An upright plain abdominal radiograph showing small bowel air-fluid levels (arrows).

The abdominal X-ray shows dilated bowel loops with air-fluid levels suggestive of obstruction (Figure 1).

**Figure 2:**
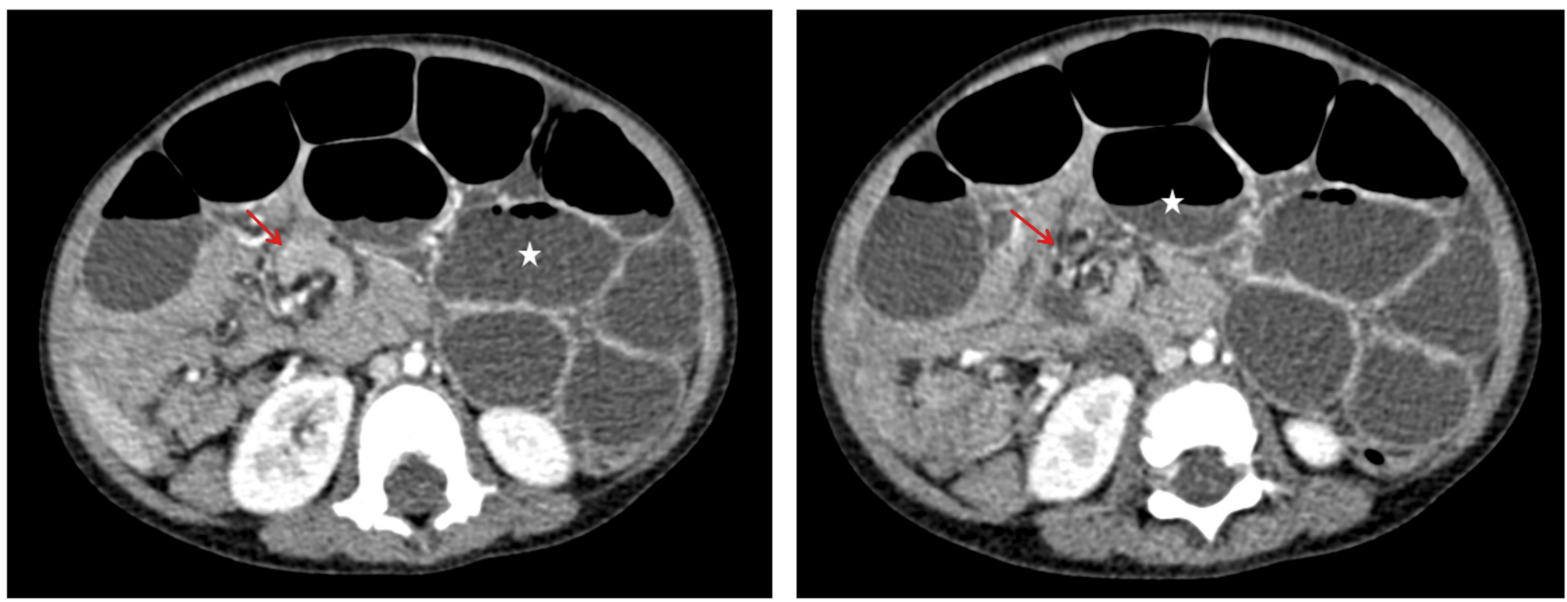
Axial slices of an abdominal CT scan showing small bowel obstruction (asterisk) upstream of a "whirlpool sign" (arrow), indicative of small bowel volvulus.

**Figure 3:**
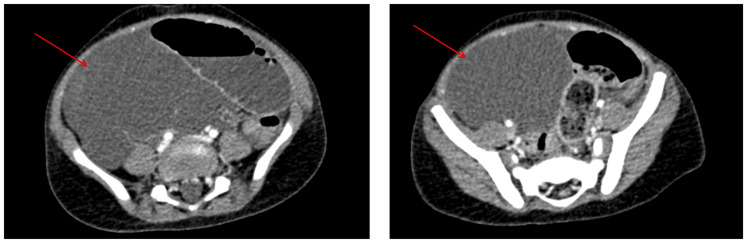
Axial slices of an abdominal CT scan showing a right-sided intra-peritoneal cystic formation displacing digestive structures to the left, without a clearly defined wall, containing fluid and without internal septations.

An abdominal ultrasound, complemented by a CT scan, revealed significant distension of the small bowel loops measuring 80 mm upstream of a "whirl sign" at the right flank, consistent with a mechanical small bowel obstruction due to volvulus (Figures 2-3). Laboratory tests were normal.

**Figure 4:**
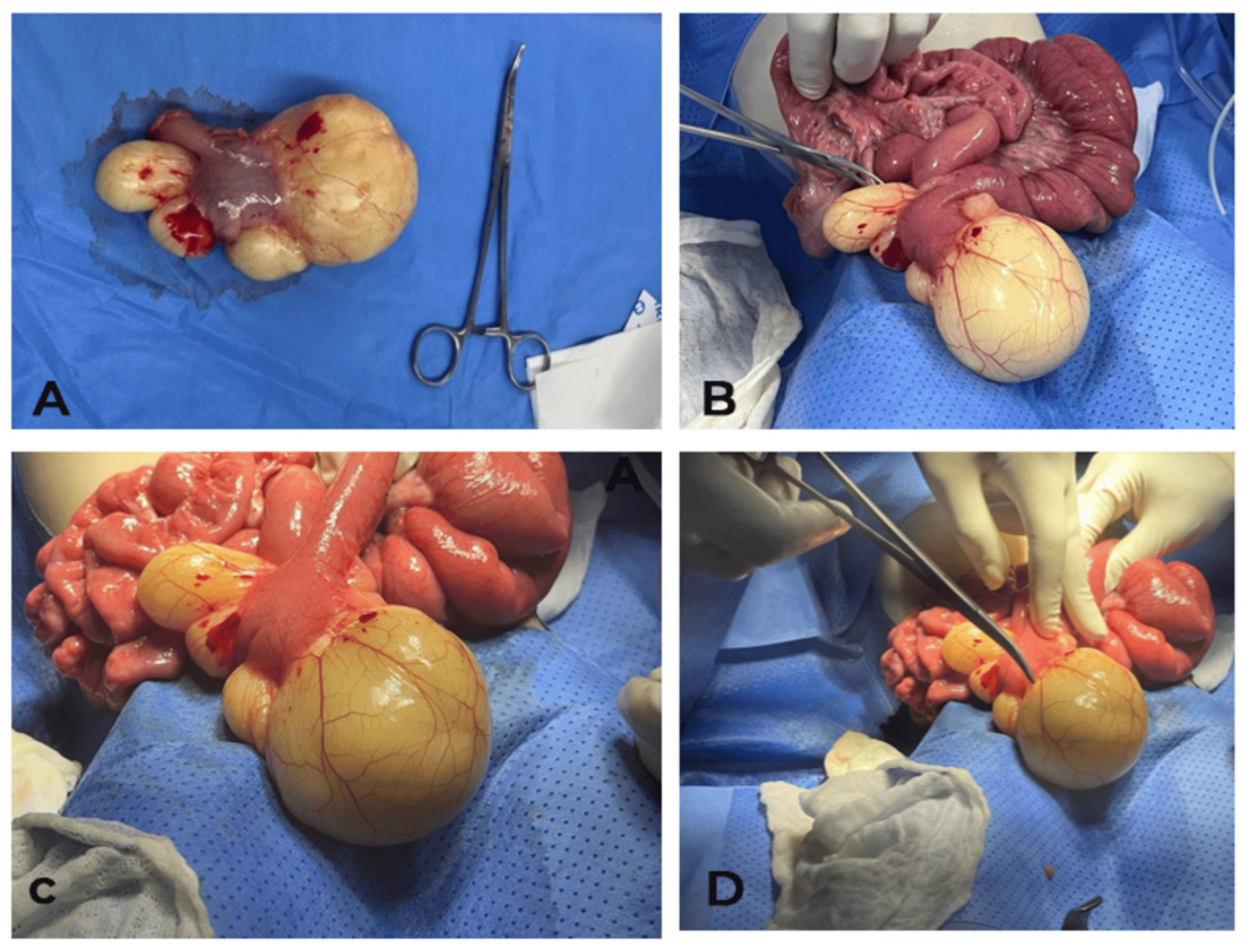
Peroperative view. A: The cyst after resection; B-C-D: Location of the lymphangioma to the Ileal loop.

The child was emergently taken to the operating room for a right sub-umbilical laparotomy. Intraoperative findings included a volvulus with significant upstream intestinal dilation but no signs of bowel ischemia. A whitish, multi-lobulated cystic mass measuring 10 cm was found adhered to the mesenteric border. The procedure involved detorsion, resection of the cyst along with a 5 cm segment of the small intestine, and a terminal anastomosis of the proximal jejunum (Figure 4). The resected specimen was sent for histopathological examination.

Postoperative recovery was uneventful. The patient was placed on antibiotics (amoxicillin + clavulanic acid), with feeding withheld for three days, maintained on standard fluids, rehydration, and close monitoring of temperature, a nasogastric tube, and bowel transit.

**Figure 5:**
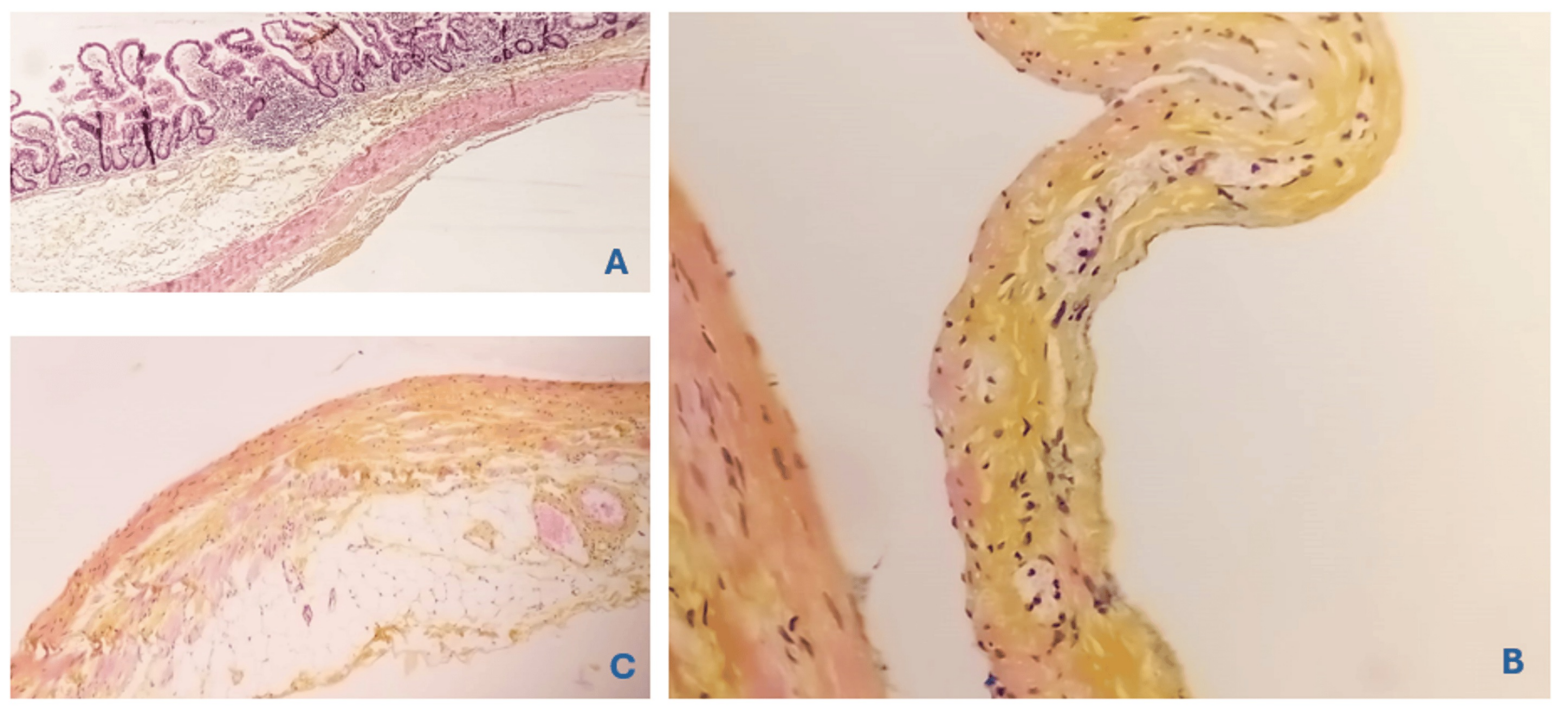
Anatomopathological image. (A) An ileal wall containing a large cavity (HES x10). (B) Lined by flattened endothelial cells and focally containing foamy histiocytes (HES x20). (C) The wall of this cavity exhibits areas with a thin layer of smooth muscle tissue (HES x20).

Histopathological examination revealed an ileal wall lined with mucosa of regular appearance, supported by fibrous tissue containing some lymphoid follicles with germinal centers. There were no signs of malignancy, consistent with a cystic subserous lymphangioma of the small intestine with clear margins (Figure 5).

Case 2

**Figure 6:**
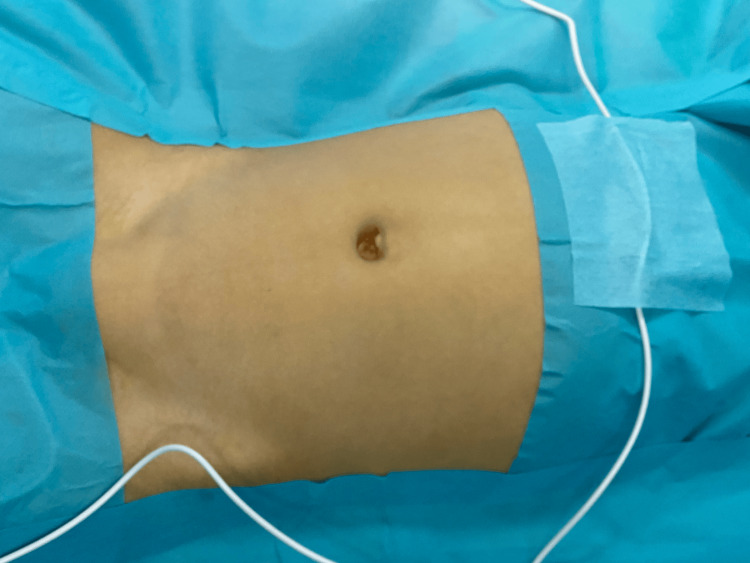
Clinical image of the abdomen.

A seven-year-old female presented with a three-day history of progressive abdominal distension (Figure 6), bilious vomiting, and constipation. The child was afebrile but exhibited signs of dehydration and abdominal tenderness.

Imaging Findings

**Figure 7:**
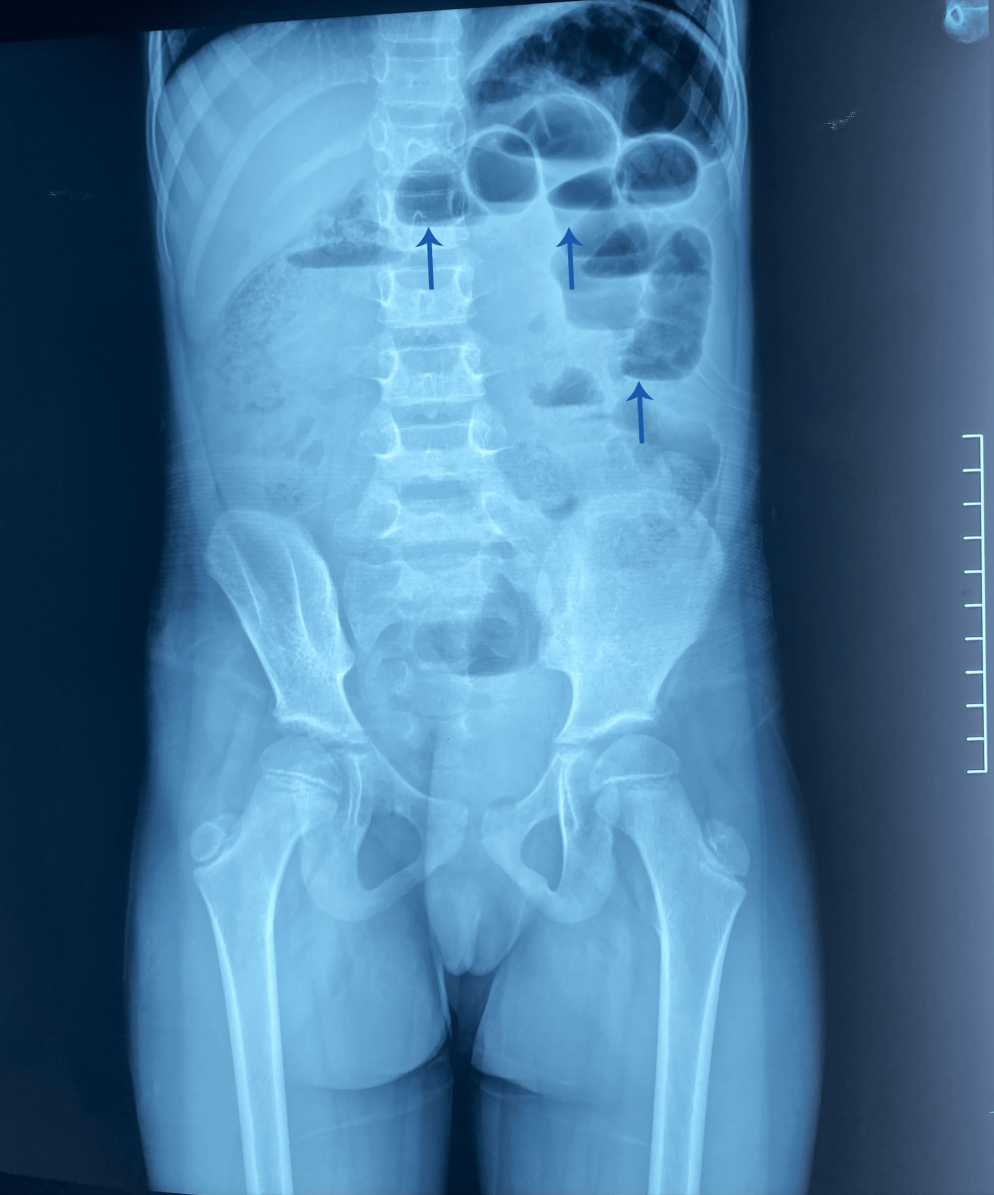
An upright plain abdominal radiograph showing small bowel air-fluid levels (arrows).

The abdominal X-ray shows gastric and colonic distension with fecal stasis (Figure 7).

**Figure 8:**
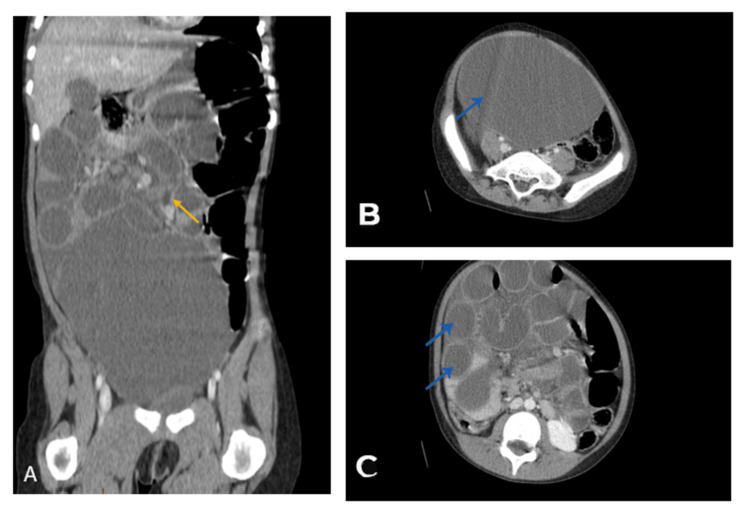
CT scan images. A: Coronal section of an abdominopelvic CT scan in the portal phase showing loops of the bowel (yellow arrow). B. Axial section of an abdominopelvic CT scan in the portal phase demonstrating an abdominopelvic mass consistent with cystic lymphangioma (blue arrow). C. Axial section of an abdominopelvic CT scan in the portal phase showing distension of intestinal loops (blue arrow).

Abdominal ultrasound and CT imaging revealed significant small bowel distension (28 mm) upstream of a whirlpool sign involving an ileal loop. A large, thin-walled intra-abdominal cystic mass (144 x 137 x 120 mm) was identified, displacing the bladder and bowel. The findings were consistent with a mechanical small bowel obstruction due to volvulus (Figure 8). Laboratory results were normal. Tumor markers were made (Table 1).

**Table 1:** Tumor marker values.

Variables	Results	Physiological values
CEA (Carcinoembryonic Antigen)	<1.73 ng/mL	<5 ng/mL
AFP (Alpha-Fetoprotein)	<2 ng/mL	<7 ng/mL
CA 125 (Cancer Antigen 125)	21.20 U/mL	<35 UI/mL
CA 19-9 (Cancer Antigen 19-9)	<2.06 U/mL	<37 UI/mL
BHCG (Human Chorionic Gonadotropin)	<1.20 mUI/mL	<1.20 mUI/mL

Surgical Intervention

**Figure 9:**
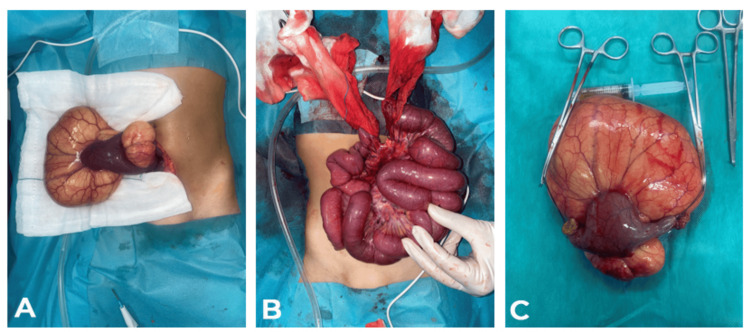
Perioperative view. A: Location of the lymphangioma to the Ileal loop. B: Image before anastomosis and after mass resection. C: Cyst after resection

The patient underwent an emergency median laparotomy. Findings included a moderate serous fluid effusion and a cystic mass (~20 cm) originating from the small bowel, located 20 cm from the Treitz ligament. A small bowel volvulus with a single turn was observed. The mass was resected, including 5 cm of the small bowel, followed by an end-to-end anastomosis and Redon drain placement (Figure 9).

Postoperative Course

Recovery was uneventful, managed with antibiotics (amoxicillin and clavulanic acid), fasting for three days, rehydration, nasogastric decompression, and close monitoring of temperature and bowel function.

Histopathology

**Figure 10:**
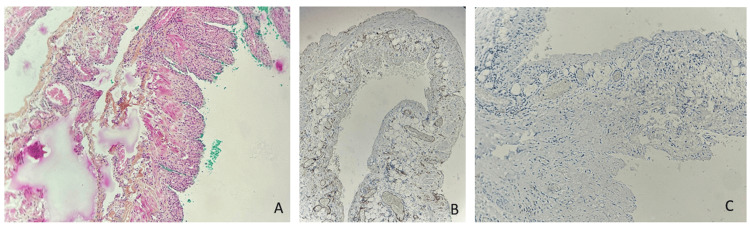
A close look at the serosal side shows confluent cavities covered in flat cells and filled with a lightly eosinophilic substance. The walls of these cavities focally hold strands of smooth muscle tissue (A). These flat cells stain with CD31 and are not positive for calretinin, evidence of their endothelial nature (B, C).

Analysis confirmed an ileal wall lined with normal mucosa, supported by fibrous tissue with lymphoid follicles, no malignancy, and findings consistent with subserosal cystic lymphangioma with clear resection margins (Figure 10).

## Discussion

Pathophysiology and clinical presentation

Cystic lymphangiomas result from the failure of embryonic lymphatic vessels to communicate with the venous system. The accumulation of lymphatic fluid leads to cyst formation. While most cases are diagnosed in early childhood, some remain asymptomatic until complications arise [4,5].

Clinical symptoms of MCL depend on size and location. Smaller cysts may be asymptomatic, whereas larger cysts can cause abdominal distension, vomiting, and obstruction. Volvulus, as seen in our cases, is a rare but serious complication requiring emergent intervention.

Diagnostic imaging

Abdominal X-rays may show non-specific findings such as gastric or colonic distension, as observed in those cases. However, ultrasound and CT imaging are more definitive in identifying the mass's cystic nature and confirming the MCL diagnosis [6]. CT scans often reveal well-defined, thin-walled cystic lesions, and the whirlpool sign observed in the second case indicates intestinal rotation anomalies [7].

Surgical management

Complete excision is the definitive treatment to prevent recurrence. Laparotomy remains the preferred approach for large, complex lesions. Laparoscopy is feasible in select cases but may be challenging for extensive cysts with bowel involvement [7,8]. Intraoperative findings of serous fluid and cystic masses displacing adjacent organs are consistent with typical MCL presentations [9].

Histopathology cystic lymphangiomas are composed of endothelial-lined lymphatic spaces filled with proteinaceous fluid. Histology confirms their benign nature and distinguishes them from other cystic lesions, such as mesenteric cysts or teratomas [10,11]. The histopathological analysis confirmed subserosal cystic lymphangioma, characterized by lymphoid follicles and fibrous tissue, with no malignancy. These findings align with previous reports, where MCL is typically benign, although recurrence can occur if resection is incomplete [4,11]. Clear surgical margins, as seen in those cases, are essential for reducing recurrence rates.

The patient’s uneventful recovery, supported by antibiotics, fasting, and rehydration, reflects standard postoperative care for bowel surgery. Most patients with MCL recover well following surgical intervention, though long-term follow-up is recommended to monitor for potential recurrence [10,12].

The post-operative course was simple in both children, with a three-year follow-up and annual ultrasound check-up.

## Conclusions

Although rare, volvulus due to cystic lymphangioma should be considered in pediatric patients presenting with acute intestinal obstruction and abdominal masses. Early diagnosis and prompt surgical intervention are essential to prevent severe complications. Complete surgical excision remains the cornerstone of treatment, with an excellent prognosis when achieved.
